# Aortic angiosarcoma leading to paraplegia: a case report

**DOI:** 10.3389/fcvm.2025.1551262

**Published:** 2025-04-29

**Authors:** Jing Li, Jiubo Sun

**Affiliations:** ^1^ Department of Traditional Chinese Medicine, Zibo Central Hospital, Zibo, Shandong, China; ^2^ Department of Oncology, Zibo Central Hospital, Zibo, Shandong, China

**Keywords:** angiosarcoma, aorta, case report, paraplegia, spinal artery

## Abstract

**Background:**

Aortic angiosarcoma is a rare disease with atypical clinical symptoms. It often initially presents with embolic phenomena, such as lower limb arterial embolism, visceral embolism, or cerebral embolism, or as renovascular hypertension.

**Methods:**

There are no reported cases in the literature of spinal cord embolism caused by this condition. We report a case of a 76-year-old female patient whose abdominal CT showed a soft tissue mass in the left hip, involving the sacrum and iliac bone.

**Results:**

Aortic CTA revealed irregular filling defects in the aortic arch and descending aorta, highly suggestive of a tumor. A biopsy of the left hip mass confirmed the diagnosis of angiosarcoma. Immunohistochemistry results were positive for Vimentin, CD31, and ERG. Based on the patient's CTA findings, a diagnosis of aortic angiosarcoma with left hip soft tissue metastasis was made. The patient developed paraplegia and lower limb arterial embolism and died shortly afterward. This article briefly reviews the literature on aortic angiosarcoma.

**Conclusions:**

The purpose of this case report is to highlight the importance of monitoring common embolic sites in clinical practice, while also considering the possibility of rare sites, such as spinal cord embolism.

## Introduction

Aortic angiosarcoma is a rare disease with a poor prognosis (1). The average age of onset is 60 years, with a higher incidence in males than in females, at a ratio of 1.59:1 (2). Clinically, it most commonly presents with embolic phenomena, such as limb ischemia, visceral embolism, cerebral embolism, or renovascular hypertension, as initial symptoms (3). There are no reported cases of spinal cord embolism caused by this disease in the literature. Imaging studies for evaluation include CT angiography (CTA), magnetic resonance angiography (MRA), and 18F-fluorodeoxyglucose positron emission tomography (18F-FDG PET/CT) (4, 5).

## Case report

This study was approved by Zibo Central Hospital (#2024Y-165, issued on September 18, 2024). The patient, a 76-year-old female, was admitted on July 2, 2024, with a left hip mass detected 2 days ago.

Medical history: 10-year history of hypertension, with a maximum blood pressure of 180/100 mmHg, treated with nifedipine sustained-release tablets, though blood pressure control was poor; No history of thoracic spinal trauma.

**Figure 1:**
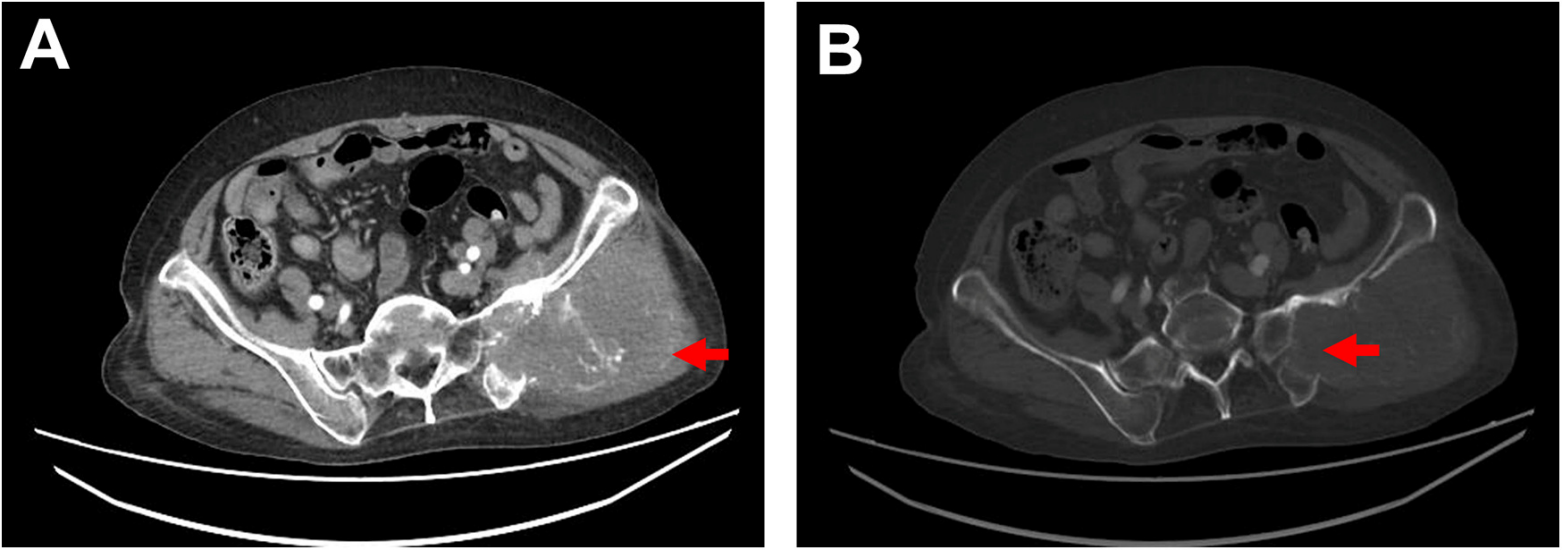
Ct imaging. **(A)** Contrast-enhanced CT shows a soft tissue mass in the left hip measuring approximately 10 cm × 9 cm, with irregular margins, locally involving the sacrum and ilium, accompanied by bony destruction of the sacrum and ilium, as indicated by the red arrow. **(B)** Bone window view displays bony destruction of the sacrum and ilium, as indicated by the red arrow.

Physical examination: The patient's general condition was stable, with a hard mass palpable in the left hip, approximately 9 cm × 9 cm in size, fixed, and tender. Blood test: D-dimer level was 6.13 mg/L. Chest and abdominal CT conducted on July 5, 2024 revealed a soft tissue mass in the left hip involving the sacrum and iliac bone, consistent with the appearance of a malignant tumor (Figures 1A,B); a filling defect in the thoracic aorta suggested a possible tumor.

**Figure 2:**
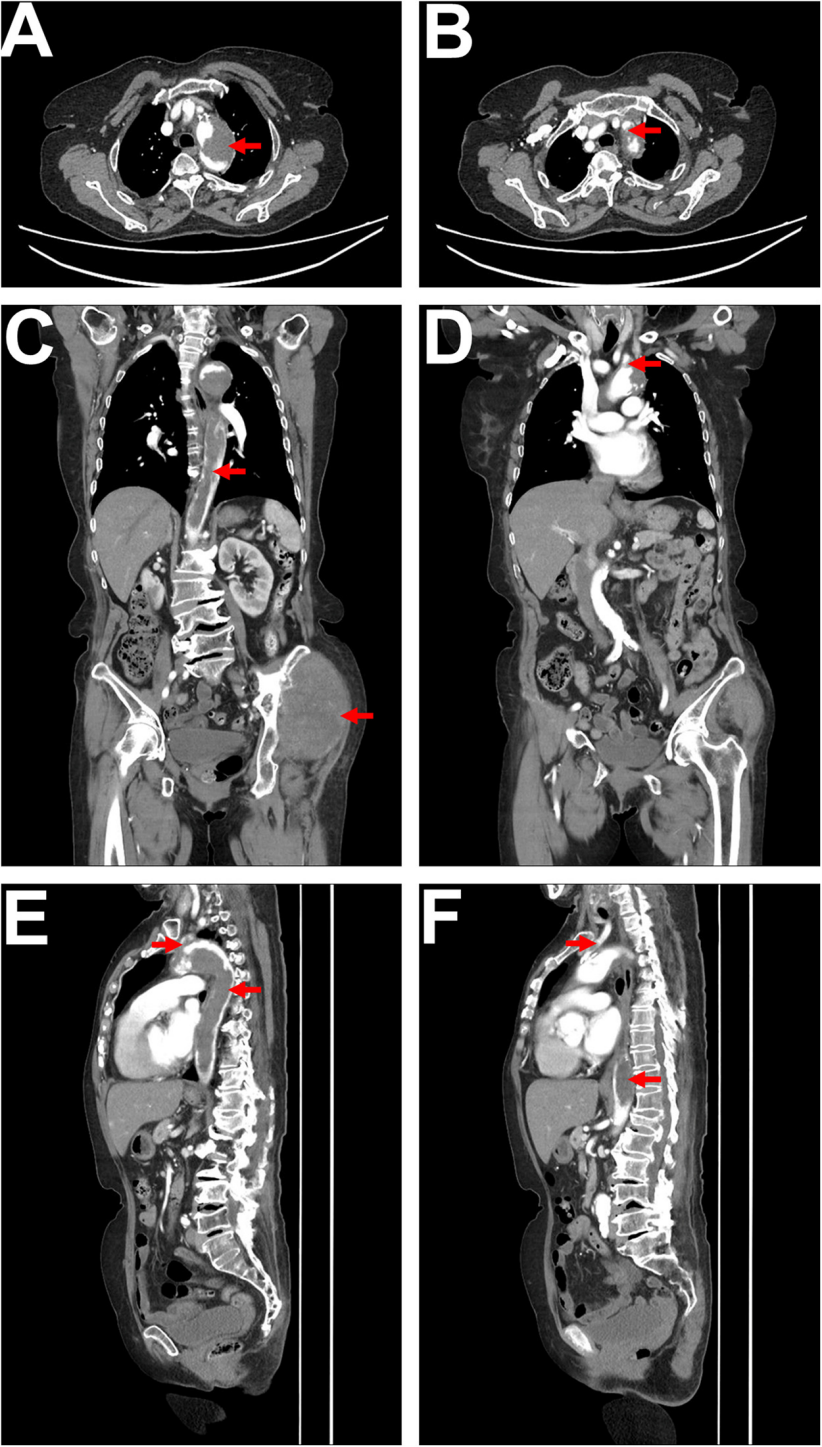
CTA imaging. **(A)** Axial CTA shows a low-density filling defect within the aortic arch, with no significant enhancement, irregular margins, and a lobulated appearance, resulting in significant lumen narrowing, as indicated by the red arrow. **(B)** Axial CTA reveals a low-density filling defect at the origin of the left subclavian artery, connected to the lesion within the aortic arch, with lumen narrowing, as indicated by the red arrow. **(C)** Coronal CTA displays a long low-density filling defect within the aorta (extending from the superior margin of the aortic arch to the level of the left renal upper pole), characterized by irregular margins and nearly complete occlusion of the lumen, as indicated by the red arrow. The soft tissue mass in the left hip is also visible, as indicated by the red arrow. **(D)** Coronal CTA shows a low-density filling defect at the origin of the left subclavian artery, connected to the lesion in the aortic arch, as indicated by the red arrow. **(E)** Sagittal CTA illustrates a long low-density filling defect within the aorta (from the superior margin of the aortic arch to the level of the left renal upper pole), with irregular margins (indicated by the red arrow), alongside a low-density filling defect at the origin of the left subclavian artery connected to the lesion in the aortic arch, as indicated by the red arrow. **(F)** Sagittal CTA shows a low-density filling defect within the left common carotid artery (indicated by the red arrow), as well as low-density lesions in the descending thoracic aorta and abdominal aorta (indicated by the red arrow).

To further clarify the aortic lesion, an aortic CTA was performed, which showed irregular filling defects in the aortic arch and descending aorta. The left subclavian artery origin and the left common carotid artery origin were also affected, with a high likelihood of tumor involvement based on the patient's medical history (Figures 2A–F). The CTA also revealed atherosclerotic changes in the aorta and its branches. Lower limb arterial ultrasound indicated bilateral iliac artery sclerosis, with arterial sclerosis and multiple plaque formations in both lower limbs. Cardiac ultrasound detected a hyperechoic mass within the descending aorta, measuring approximately 31 × 25 mm, with a relatively clear boundary, irregular shape, minimal movement, and localized lumen narrowing.

Mild intimal thickening (∼1.0 mm) and multiple mixed-echo plaques were present in the lower extremity arteries, with the largest plaques in the common femoral arteries (right: 10.1 × 2.8 mm, left: 8.8 × 2.1 mm), but no significant stenosis or dilation was observed.

**Figure 3:**
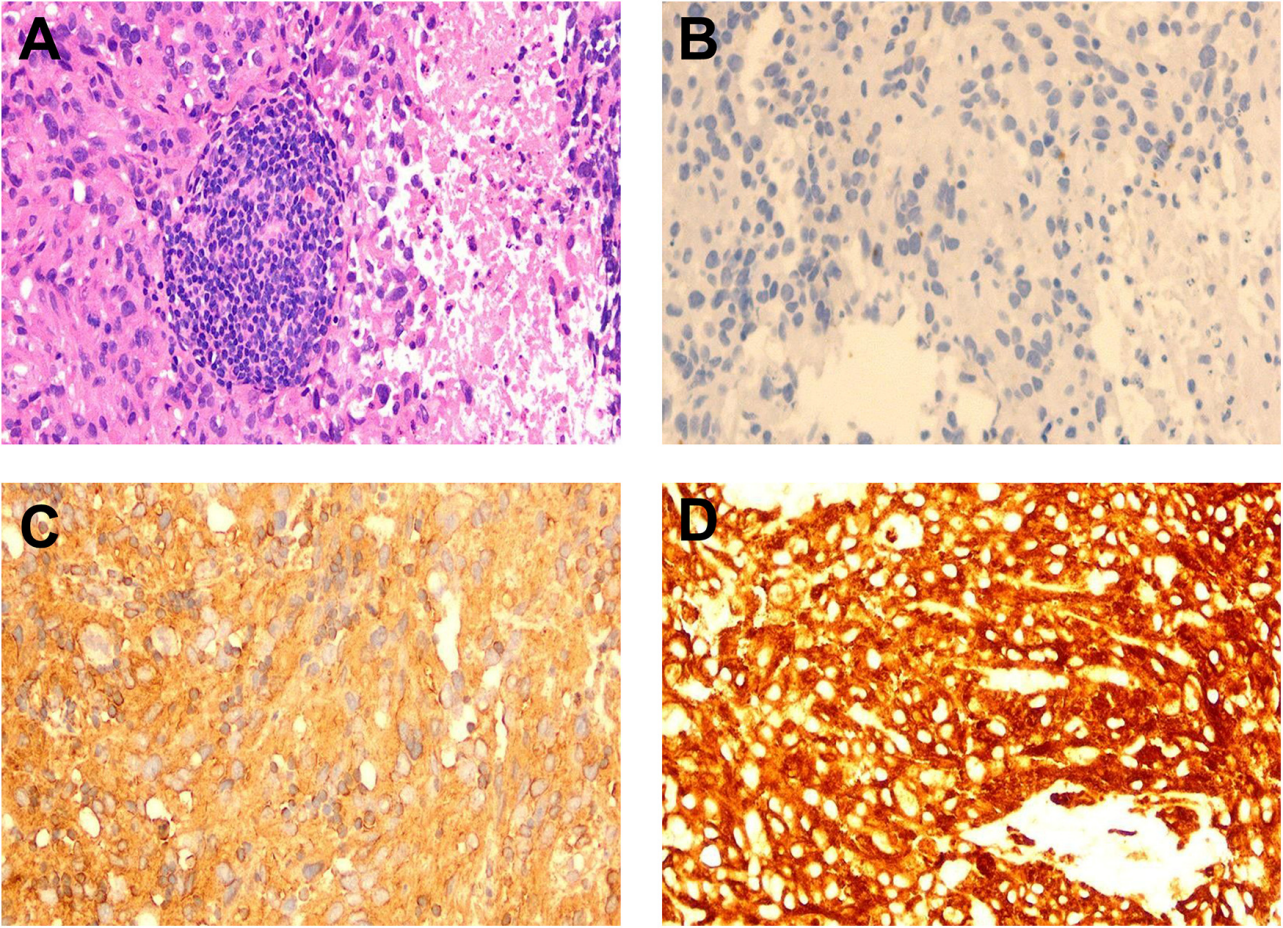
Pathology and immunohistochemistry staining. **(A)** Pathological examination of the left hip mass biopsy reveals angiosarcoma with low tumor differentiation, showing plump spindle-shaped cells, and features such as nucleoli, mitosis, and sheet-like necrosis (HE staining, ×200). **(B)** Immunohistochemistry for CKAE1/CKAE3 shows negative results (-) (×200). **(C)** Immunohistochemistry for Vimentin shows positive results (+) (×200). **(D)** Immunohistochemistry for CD31 shows positive results (+) (×200).

Due to the risk of embolism associated with an intravascular biopsy of the aorta, a biopsy of the left hip mass was performed instead. Pathology indicated angiosarcoma with multifocal necrosis (Figure 3A). Immunohistochemistry results were as follows: CKAE1/AE3 (-); Vimentin (+); CD31 (+); ERG (+); KI-67 (+) with a 70% proliferation index (Figures 3B–D). Based on the imaging findings and a multidisciplinary consultation, the diagnosis was confirmed as aortic angiosarcoma with metastasis to the left hip soft tissue.

The patient and her family refused anti-tumor treatment. She was given oral rivaroxaban 20 mg once daily for anticoagulation, along with palliative and symptomatic care. On July 6, 2024, at 23:00, the patient suddenly developed bilateral lower limb paralysis and urinary and fecal incontinence. Physical examination revealed 0/5 muscle strength in both lower limbs, loss of deep and superficial sensation below the umbilicus, significantly decreased skin temperature in both legs, and absence of bilateral femoral pulses. She was diagnosed with paraplegia (due to T10 spinal cord injury) and lower limb arterial embolism. The patient's condition rapidly deteriorated, and she passed away on July 7, 2024, at 19:00.

## Discussion

Primary aortic sarcoma is extremely rare. Brodowski first described this disease in 1873. As of 2019, fewer than 250 cases have been reported in the literature (2). Based on the location of the aortic sarcoma relative to the aortic wall, it is divided into intimal and wall types. The intimal type can be further subdivided into intimal angiosarcoma and intimal myofibroblastoma, while the wall type histologically originates from the media or adventitia and expresses mesenchymal-specific antigens in immunohistochemistry (6). In aortic sarcomas, 80% are intimal type, while the remaining 20% are wall type (7). Aortic sarcoma can occur in all segments of the thoracic and abdominal aorta, with an incidence of 42.6% in the abdominal aorta, 37.7% in the thoracic aorta, 7.6% in the aortic arch, and 12.1% in both thoracic and abdominal segments (2).

The clinical symptoms are closely related to the tumor's location and growth pattern. The intimal type grows along the intima within the lumen and can form polypoid masses, which may include pedunculated floating portions that can move freely within the aortic lumen during each cardiac cycle. Twenty percent of patients may experience acute ischemic events due to embolization of tumor fragments (8). Symptoms may include intermittent claudication, mesenteric artery occlusion, occlusion-related hypertension, and sexual dysfunction (7). Distal embolization leading to occlusion at the arterial ends can result in gangrene. For example, mesenteric artery embolism can lead to intestinal infarction (9), renal artery embolism can lead to renal infarction (10), and lower limb artery embolism can lead to lower limb gangrene (10). These tumors can also produce metastatic tumor emboli, with bone, lung, liver, and skin being the most common sites of metastasis (11). Up to 56% of patients have distant metastasis at initial diagnosis (2). Wall-type aortic sarcomas grow outward, primarily affecting the media and adventitia, and typically do not obstruct blood vessels. As the lesions progress, they usually present with systemic cachexia symptoms such as fever, weight loss, and anorexia.

Aortic angiosarcoma can lead to the formation of tumor emboli. These emboli may dislodge and travel through the bloodstream, potentially lodging in the spinal vasculature. The lodgment of these emboli can cause occlusion of spinal arteries, leading to ischemia and infarction of spinal cord tissue. This process is particularly concerning given the spinal cord's reliance on a delicate network of arterial supply, including the anterior and posterior spinal arteries (12). Beyond tumor embolization and vascular occlusion, several factors can exacerbate the risk of spinal cord ischemia in patients with aortic angiosarcoma, including surgical interventions, collateral circulation disruption, hemodynamic instability.

Imaging studies used for evaluation include CT angiography (CTA), magnetic resonance angiography (MRA), and 18-fluorodeoxyglucose positron emission tomography (18F-FDG PET/CT) (4, 5). The CT characteristics of aortic sarcoma include prominent intraluminal growth, irregular surface margins, lobulated or mildly lobulated appearance, minimal or absent contrast enhancement, thickening and/or enhancement of the aortic wall, extravascular extension, and lymph node or distant metastasis (4, 5, 7). The patient we reported had intimal angiosarcoma affecting the aortic arch, descending thoracic aorta, and abdominal aorta, with CT showing prominent intraluminal growth, irregular surface margins, slight lobulation, and minimal contrast enhancement, along with soft tissue metastasis in the hip, consistent with findings reported in the literature (4, 5, 7). Contrast-enhanced and perfusion MRI can suggest tumor formation (7). MRI has been shown to be superior to CT in differentiating tumors from the vascular wall and surrounding soft tissues (9). Previous literature reports that almost all aortic sarcomas exhibit metabolic activity on 18F-FDG PET, which can better differentiate them from metabolically inactive atherosclerosis, intraluminal thrombus, and chronic aortic dissection (13).

The gold standard for the pathological diagnosis of aortic angiosarcoma is tissue biopsy; however, intra-arterial biopsy is rarely performed due to the potential for embolic complications. Many cases are definitively diagnosed through the resection or biopsy of metastatic lesions (14–16). In this case, to avoid complications such as embolism from intra-arterial puncture biopsy, a biopsy of the left hip mass was performed, which confirmed the diagnosis of angiosarcoma. Immunohistochemistry results were as follows: CKAE1/AE3 (-); Vimentin (+); CD31 (+); ERG (+); KI-67 (+) with a proliferation index of 70%. Based on the patient's CTA findings, the diagnosis was established as aortic angiosarcoma with metastasis to the left hip soft tissue, similar to a reported case by Won Jong Bahk et al. (16).

The most effective treatment for aortic angiosarcoma is radical surgery, which involves resection of the affected portion of the aorta followed by replacement (17). If surgical resection is not feasible, palliative measures such as endarterectomy, bypass surgery, stent placement, and localized radiotherapy can be used to relieve symptoms (18). The prognosis for aortic sarcoma is poor, with a median survival of only 8 months; the 1-year, 3-year, and 5-year survival rates are 26%, 7.6%, and 3.5%, respectively (2). Studies have shown that the median survival of patients undergoing surgery combined with chemotherapy is significantly better than that of those receiving chemotherapy alone or surgery alone (surgery-chemotherapy, 12 [8–24] months; chemotherapy alone, 8 [5–10] months; surgery alone, 7 [2–16] months; no treatment, 2 [0.5–15] months; *P* = 0.001) (2). Angiosarcoma may possess a unique ability to promote pathological coagulation through direct endothelial damage, intraluminal obstruction, relative hemostasis, and cytokine modulation (19). Approximately 20% of patients will experience arterial embolic events (8). Patients without contraindications to anticoagulation should be given anticoagulant therapy alongside anti-tumor treatment (18, 20). However, anticoagulant therapy does not prevent the detachment of emboli (20), as evidenced by this patient, who experienced spinal cord embolism and lower limb arterial embolism despite being on oral rivaroxaban.

In this case, the angiosarcoma affected the aortic arch, descending thoracic aorta, abdominal aorta, as well as the left common carotid artery and left subclavian artery arising from the aortic arch. The aortic CTA showed intimal lesions with significant intraluminal growth and irregular surface margins. The patient experienced lower limb arterial embolism and developed paraplegia, with the injured spinal cord segment being the T10 thoracic segment.

The sudden onset of paraplegia in this patient is quite rare. Previous tests did not support spinal fractures or dislocations, external spinal cord compression, spinal cord metastasis as the causes. CTA showed involvement of the left subclavian artery, which supplies the vertebral artery, embolism of the spinal artery due to an embolus dislodging is a possible explanation. The thoracic spinal cord is the longest segment of the spinal cord, with a relatively poor blood supply, making it more susceptible to pathological changes. We hypothesize that the paraplegia in this patient was caused by embolism of the spinal artery due to involvement of the left subclavian artery. Although spinal cord MRI could identify the specific segment of spinal cord embolism, and spinal angiography could determine the affected blood vessels, the family refused further examination. This is the limitation of our report. Due to the massive size of the aortic tumor and the presence of soft tissue metastasis, the patient passed away quickly, and the family declined an autopsy, preventing further clarification of the specific embolized vessels.

## Data Availability

The original contributions presented in the study are included in the article/Supplementary Material, further inquiries can be directed to the corresponding author.
